# Highly Tunable MOCVD Process of Vanadium Dioxide Thin Films: Relationship between Structural/Morphological Features and Electrodynamic Properties

**DOI:** 10.3390/s23167270

**Published:** 2023-08-19

**Authors:** Anna Lucia Pellegrino, Francesca Lo Presti, Gian Paolo Papari, Can Koral, Antonello Andreone, Graziella Malandrino

**Affiliations:** 1 Dipartimento di Scienze Chimiche, Università di Catania, and INSTM UdR Catania, Viale A. Doria 6, I-95125 Catania, Italy; annalucia.pellegrino@unict.it (A.L.P.); francesca.lopresti@phd.unict.it (F.L.P.); 2 Dipartimento di Fisica “E. Pancini”, Università di Napoli “Federico II”, Via Cinthia, I-80126 Napoli, Italy; gianpaolo.papari@unina.it (G.P.P.); antonello.andreone@unina.it (A.A.); 3 Naples Research Unit, Institute for Superconducting and Other Innovative Materials and Devices (SPIN), Consiglio Nazionale delle Ricerche (CNR), Via Cinthia, I-80126 Napoli, Italy; 4 Naples Division, Istituto Nazionale di Fisica Nucleare (INFN), Via Cinthia, I-80126 Napoli, Italy; can.koral@unibas.it; 5 Department of Science, University of Basilicata, Viale dell’Ateneo Lucano 10, I-85100 Potenza, Italy

**Keywords:** monoclinic structure, X-ray powder pattern, THz properties

## Abstract

The monoclinic structures of vanadium dioxide are widely studied as appealing systems due to a plethora of functional properties in several technological fields. In particular, the possibility to obtain the VO_2_ material in the form of thin film with a high control of structure and morphology represents a key issue for their use in THz devices and sensors. Herein, a fine control of the crystal habit has been addressed through an in-depth study of the metal organic chemical vapor deposition (MOCVD) synthetic approach. The focus is devoted to the key operative parameters such as deposition temperature inside the reactor in order to stabilize the P2_1_/c or the C2/m monoclinic VO_2_ structures. Furthermore, the compositional purity, the morphology and the thickness of the VO_2_ films have been assessed through energy dispersive X-ray (EDX) analyses and field-emission scanning electron microscopy (FE-SEM), respectively. THz time domain spectroscopy is used to validate at very high frequency the functional properties of the as-prepared VO_2_ films.

## 1. Introduction

Vanadium oxides have attracted growing interest in the last decades due to their unique electrical, optoelectronic and magnetic properties [1,2]. The peculiar multi-oxidation states of vanadium, ranging from +2 to +5, are reflected in various crystalline structures and thus highly tunable functional properties [3,4]. Particularly, four vanadium oxides are characterized by single oxidation states (+2 for VO, +3 for V_2_O_3_, +4 for VO_2_ and +5 for V_2_O_5_), and others have mixed oxidation states, resulting in a plethora of different V-O ratios. The polymorphism of vanadium oxide, characterized by crystalline structures with different oxygen coordination, results in the formation of octahedral, pentagonal bipyramid, square pyramid and tetrahedral sharing corners, edges or faces [1,5]. Consequently, the multi-oxidation state of the vanadium cations and the various crystalline structures dramatically affect the physical and chemical properties of the final materials [5]. Particularly, the vanadium oxides exhibit excellent intercalation properties [6,7], catalytic activities [8,9], outstanding phase transitions (metal−insulator transition) [10,11] and high electrical conductivity [12]. The above-mentioned features have opened the way for recent research to explore the potential of vanadium oxide materials in ion batteries, water splitting, smart windows, supercapacitors, sensors and in reconfigurable metasurfaces [13,14,15,16,17,18].

Among the different V-O structures, several VO_2_ crystal phases are known, including tetragonal VO_2_(R) (P4_2_/mnm), monoclinic VO_2_(M) (P2_1_/c), tetragonal VO_2_(A) (P4_2_/nmc), monoclinic VO_2_(B) (C2/m) and triclinic VO_2_(T) (P*(2)) [19]. Recently, monoclinic structures of vanadium dioxide have attracted growing interest due to their unique optical and electrical properties useful in several technological applications, such as supercapacitors, sensors and metasurfaces at very high frequency. In particular, VO_2_(M) and VO_2_(B) structures have attracted great attention due to their unique sensing activities [20]. Recently, we tested the interesting sensing properties of vanadium dioxide VO_2_(M) P2_1_/c phase through the fabrication of an open cavity operating in the THz regime. Particularly, the cavity has been realized by employing as mirrors two VO_2_ thin films grown on silicon parallel supports. The effect of a variable length of the cavity and the transition between insulating and conducting states have been evaluated [21].

However, the massive application of V-O phases as key materials in the field of sensors and active metasurfaces for the manipulation of the electromagnetic (EM) fields requires the development of synthetic strategies which allow a high degree of uniformity in both thickness and composition over large areas for the fabrication of thin films. Among the different synthetic strategies tested in literature, pulsed laser deposition [22], molecular beam epitaxy [23], magnetron sputtering [24], atomic layer deposition (ALD) [25] and electrodeposition [26,27] have been reported. Metal-organic chemical vapor deposition (MOCVD) represents, up to now, one of the most appealing approaches for the deposition of V-O films, due to its advantages, ranging from reliable and reproducible methods, fast and industrially scalable production and high control of thin film growth in terms of composition and morphology over large areas [28,29].

In the present work, we have focused our attention on the VO_2_ phase structures, due to their unique optical and electrical properties, which can be exploited to realize metadevices at very high frequency. A few examples of possible applications include sensing [1,20], beam steering [30,31], polarization control [32] and vortex beam generation with different topological charges [33].

We report an optimized MOCVD process for the selective fabrication of monoclinic VO_2_(M) P2_1_/c and/or VO_2_(B) C2/m phase structures in the form of thin films on silicon substrate starting from the [VO(acac)_2_] (bis(penta-2,4-dionato)oxovanadium) precursor. Among the various operative parameters involved in the growth, the effect of the deposition temperature has been the focus of the synthetic strategy in order to finely tune not only the crystalline structure of the VO_2_ films, but also the morphology of the film surfaces and the thickness of the materials. Furthermore, using THz time domain spectroscopy, we will show that the structural and morphological features of deposited films are strictly related to the conducting properties of the metallic-like phase, triggered by heating the vanadium oxide film to temperatures above its critical value $T_{c}=$ 78 °C [34,35].

## 2. Materials and Methods

### 2.1. Synthesis of VO_2_ Thin Films

The vanadyl-acetylacetonate [VO(acac)_2_], used as vanadium precursor, was purchased from STREM Chemicals and used without any further purifications. V-O films were deposited on Si (001) substrates in a horizontal, hot-wall MOCVD reactor in the 300–450 °C deposition temperature range, whereas the V source temperature was controlled independently and kept at 170 °C. Each section was monitored using K-type thermocouples with ±1 °C accuracy. The deposition processes were carried out under O_2_ flow at 150 sccm used as reacting gas, and Ar flow at 150 sccm as carrier gas. Both the gas flows were controlled using MKS 1160 flow controller units. The time of the process was kept at 1 h and the pressure inside the reactor at the value of 4 Torr through a scroll pump unit monitored using MKS Baratron 122AAX.

### 2.2. Characterization

An in-depth study on the structural characterization was performed through X-ray diffraction (XRD) analysis using a Smartlab Rigaku diffractometer in grazing incident mode (0.5°) operating at 45 kV and 200 mA, equipped with a rotating anode of Cu K_α_ radiation. The morphological characterization was conducted using a Field Emission Scanning Electron Microscope FE-SEM ZEISS SUPRA 55 VP. The EDX spectra were recorded using an INCA-Oxford windowless detector, having a resolution of 127 eV as the full width at half-maximum (FWHM) of the Mn K_α_ radiation. THz spectroscopy was operated in time domain by employing a commercial setup (TERAK-15 by Menlo Systems^®,^ Martinsried, Germany). THz waves were coherently generated and detected through photo-conducting antennas enabling to investigate the EM response in the range 0.1–4.0 THz with a resolution of a few GHz. Experiments were performed in a purging box filled with N_2_ gas to prevent absorption of humidity. V-O films were investigated in transmission configuration by the use of a customized sample holder enabling to control the sample temperature from room temperature (RT) up to about 400 °C. Herewith, the analysis was performed by using only two temperatures: 22 °C, representing the room temperature RT, and 90 °C when films were completely within the metallic-like phase. The THz system enables to record the time dependent electric field signals propagating through both the free space $E_{r}(t)$ and the sample $E_{s}(t)$, represented by the V-O film deposited on Si substrate. Both signals were then processed through fast Fourier transform to obtain the transmission function $\tilde{T}\left(\omega \right)={\tilde{E}}_{s}(\omega )/{\tilde{E}}_{r}(\omega )$ that depends on the complex refractive index of both substrate ${\tilde{n}}_{s}\left(\omega \right)=n_{s}\left(\omega \right)+ik_{s}(\omega )$ and thin film sample ${\tilde{n}}_{f}\left(\omega \right)=n_{f}\left(\omega \right)+ik_{f}(\omega )$. In order to improve the accuracy in the estimation of refractive index [36], the VO_2_ films were deposited only over half the area of substrates 2cm×1cm in size. By applying an in-house MATLAB^®^ code based on a total variation technique [37], ${\tilde{n}}_{f}$ was retrieved and used to calculate the complex dielectric function ${\tilde{\varepsilon }}_{f}=\varepsilon_{f}^{\prime }+i\varepsilon_{f}^{\prime \prime }$, where $\varepsilon_{f}^{\prime }=n_{f}^{2}-k_{f}^{2}$ and $\varepsilon_{f}^{\prime \prime }=2n_{f}k_{f}$. Finally, the film conductivity was evaluated by using $\sigma =2\varepsilon_{0}\omega \varepsilon_{f}^{\prime \prime }$ where $\varepsilon_{0}$ is the vacuum permittivity (8.854189 × 10^−12^ F m^−1^).

## 3. Results and Discussion

**Figure 1:**
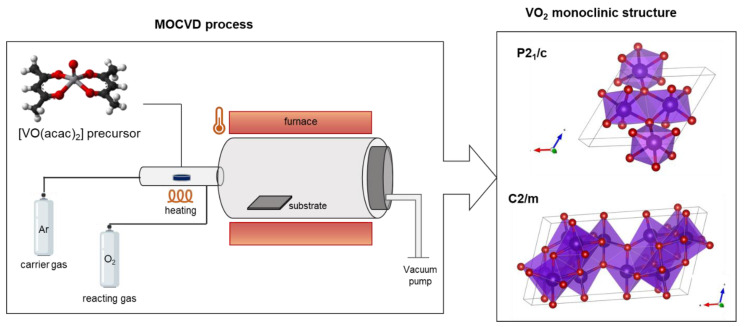
Scheme of the MOCVD process for the synthesis of VO_2_ thin films, and P2_1_/c and C2/m monoclinic structures derived through the Vesta program.

The metalorganic compound [VO(acac)_2_] has been successfully applied as a volatile and stable precursor for the fabrication of VO_2_ thin film through a MOCVD approach. The deposition temperature has been set up in the range of 300–450 °C (see Figure 1). Under the present conditions, it is possible to finely tailor the stabilization of the monoclinic P2_1_/c and/or the C2/m phases [38,39]. Even though the deposition temperature is the key parameter that has the main effect on the structure of VO_2_ films, the stabilization of the monoclinic structure as P2_1_/c and/or the C2/m has been observed to affect both the morphology and the thickness of the synthesized films. The other parameters, such as deposition time, oxygen and Ar flows and precursor source temperature, have been instead fixed at 60 min, 150 sccm and 170 °C, respectively. The results reported are well framed within the data previously reported in ref. [28]. As widely studied in the past decades, the vanadium oxide phases present different V-O ratios, which results in the formation of VO_2_, V_2_O_5_, V_4_O_9_ and V_6_O_13_ structures with different V oxidation states. In particular, the present study has focused on the stabilization of the pure monoclinic phase in the form of P2_1_/c space group due to the interesting properties they show in the THz region. In addition, the effect of changing in morphology and thickness has been analyzed and studied in relation to the functional properties of the films.

### 3.1. Structural Characterization

**Figure 2:**
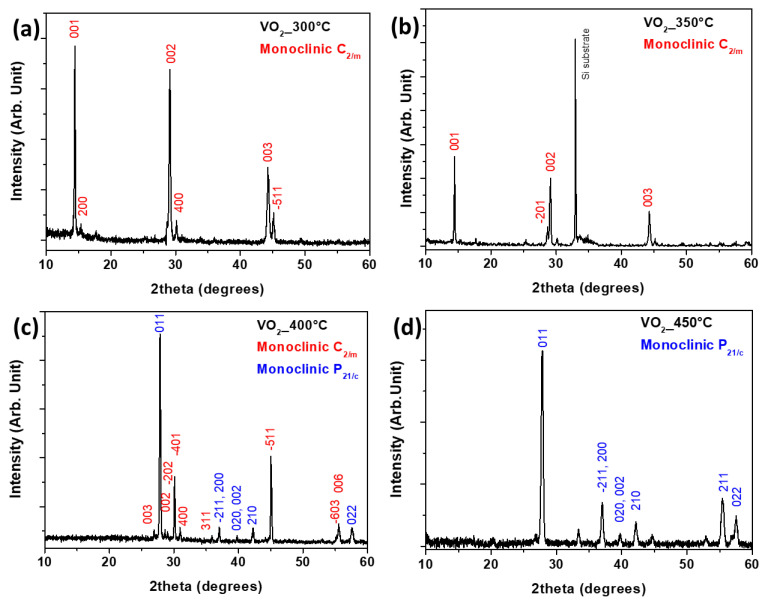
XRD patterns of VO_2_ thin films grown on Si (001) substrate at different deposition temperatures: (**a**) 300 °C, (**b**) 350 °C, (**c**) 400 °C and (**d**) 450 °C.

A structural investigation on the VO_2_ thin films obtained as a function of the different deposition temperatures has been conducted through XRD analysis. In order to assess the direct effect of this key parameter on the phase stabilization, the other process parameters have been kept constant. The pattern of the VO_2_ thin film obtained at 300 °C is reported in Figure 2a. The analysis points to the formation of a crystalline film in the form of pure monoclinic C2/m, as confirmed by comparison with PDF card n. 31-1438 and 43-1050. Particularly, the peaks at 14.46°, 29.08° and 44.20° associated with 001, 002 and 003 reflections, respectively, point to the growth of a nanostructured system with a preferential orientation along the 00ℓ. Similarly, the pattern associated with the film deposited at 350 °C (Figure 2b) shows the formation of the pure monoclinic C2/m, with the same preferential orientation along the 00ℓ. At the higher deposition temperature, i.e., 400 °C (Figure 2c), it is evident the formation of a crystalline film which has peaks associated with both the C2/m and P2_1_/c phases, as confirmed by matching with the PDF cards n. 31-1438 and 43-1050 for the first structure, and PDF cards n. 43-1051 and 44-0252 for the second one. Finally, the system synthesized at 450 °C shows the stabilization of the pure and crystalline monoclinic P2_1_/c phase (PDF file numbers 43-1051, 44-0252), as confirmed by the presence of the peaks at 27.87°, 37.08°, 39.64°, 42.18°, 55.44° and 57.52° (Figure 2d). Interestingly, thanks to the here-reported structural XRD characterization, it has been possible to identify three temperature windows, in which each monoclinic structure, i.e., C2/m and P2_1_/c, is stable as a pure phase or as a mixture, under specific operative conditions. Specifically, the deposition temperature of 400 °C represents the turning point of crystalline arrangement change. It is worth noting the orientation along the 00ℓ of the films fabricated at 300 °C and 350 °C, which represents an added value of the present MOCVD synthesis considering the low temperatures required for this thermal-induced growth process.

### 3.2. Morphological and Compositional Characterization

**Figure 3:**
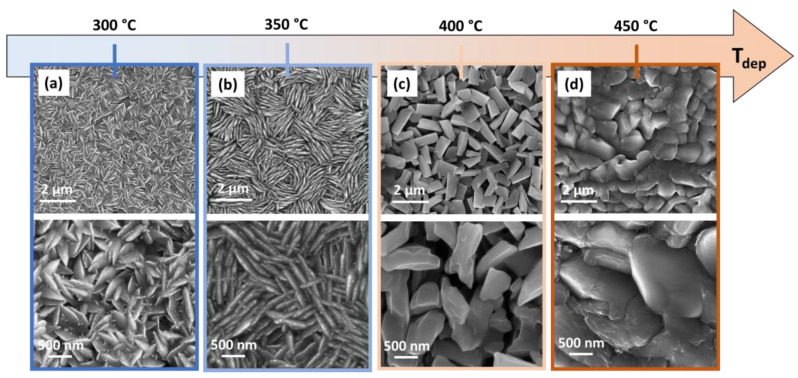
FE-SEM plan view images of VO_2_ thin films grown on Si (001) substrate at different deposition temperatures: (**a**) 300 °C, (**b**) 350 °C, (**c**) 400 °C and (**d**) 450 °C.

Morphological features of the VO_2_ films have been deeply studied by field emission scanning electron microscopy (FE-SEM). In Figure 3a, the film obtained at 300 °C deposition temperature displays the growth of compact and homogenous nanostructured film with plate-like grains characterized by regular dimensions having width in the order of 500–700 nm, mainly placed perpendicular to the substrate. The film synthesized at 350 °C, shown in Figure 3b, presents a uniform coverage of the surface with a very peculiar morphology. In fact, the film displays thinner and even more regular plate-like grains of 700–1000 nm in width, which are intertwined to form a very regular texture. For both these films, the morphology finds counterpart in the XRD patterns indicating highly oriented samples. Different from the first two morphologies, in Figure 3c, the sample obtained at 400 °C shows the formation of a homogeneous film composed of parallelepiped grains. Conversely from the other films, in this case, the system is characterized by an enhanced porosity, even though, also in this case, the homogeneity of the deposit is guaranteed over large substrate areas (up to 2 × 2 cm^2^). Finally, the VO_2_ film synthesized at 450 °C (Figure 3d) displays the formation of flake-like grains of the order of 1 µm overlapped on top of each other forming a very compact deposit with equiaxial grains. Notably, the deposition temperature affects in a significant way the morphological features of the films, which pass from a well-organized plate-like structure placed in a perpendicular way with respect to the substrates, to parallelepiped grains and to equiaxial grains. The differences can be likely attributed to two main aspects of the synthesis: (i) the balance between nucleation and growth process, which in general affects the dimensions of the grains; and (ii) the stabilization of the monoclinic structures, which pass from the C2/m structure at 300 °C and 350 °C (Figure 3a,b), to a mixture of C2/m and P2_1_/c at 400 °C (Figure 3d), and to a pure P2_1_/c at 450 °C (Figure 3d). The more porous structure of films deposited at 400 °C may be likely associated with the faster growth rate, yielding larger grains not well coalesced, and the presence of the two phases.

**Figure 4:**
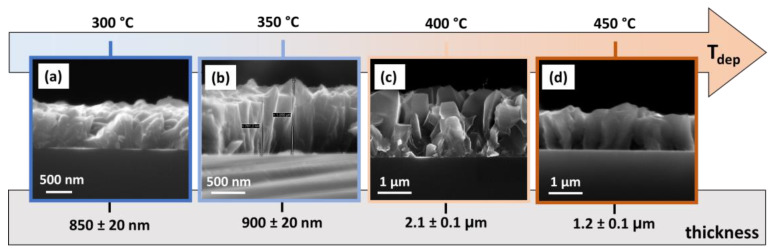
FE-SEM cross-view images of VO_2_ thin films grown on Si (001) substrate at different deposition temperatures: (**a**) 300 °C, (**b**) 350 °C, (**c**) 400 °C and (**d**) 450 °C.

Finally, the thickness evaluation of the VO_2_ samples has been conducted through FE-SEM analysis in cross-sectional mode. In Figure 4, an overview of all the four systems discussed above has been reported regarding the related thickness of the films. Notably, the thickness of the systems passes from around 850 nm and 900 nm for the samples deposited at 300 °C and 350 °C (see Figure 4a,b), to a thickness of 2.1 μm for the film at 400 °C (Figure 4c), and a value of 1.2 μm for the film at 450 °C (Figure 4d). In this case, the trend observed can be associated not only with the mere effect of the deposition temperature, but also with the different C2/m and P2_1_/c phase structure growth. Specifically, the growth rate varies from 14, 15, 35 and 20 nm/min for 300, 350, 400 and 450 °C, respectively. Finally, it is worth noting that the cross-sectional image of the film obtained at 400 °C also confirms the formation of a more porous sample, as suggested from the plan view in Figure 3c.

**Figure 5:**
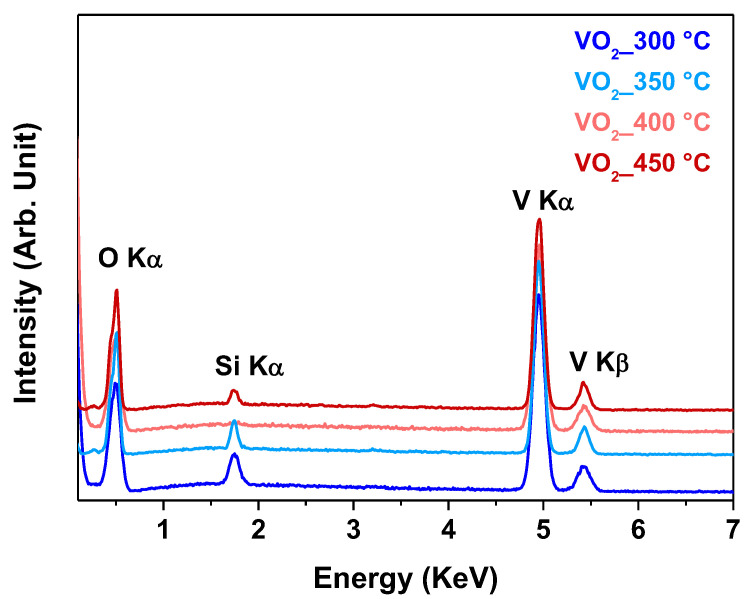
EDX spectra of VO_2_ thin films grown on Si (001) substrate at different deposition temperatures.

In addition, the compositional purity of the VO_2_ films has been assessed through energy dispersive X-ray (EDX) analyses. The EDX spectra reported in Figure 5 are quite similar and confirm the formation of pure vanadium oxide phases for all the films deposited in the range 300–450 °C. In particular, the signals found at 4.9 and 5.4 keV are associated with the K_α_ and K_β_ peaks of V, and the one found at 0.51 keV is related to the O K_α_ peak. In addition, the peak found at 1.73 keV is due to the Si K_α_ peaks of the Si (001) substrate. The different intensities of this last signal in the three samples are related to the difference in thickness of the analyzed films. Notably, the absence of signals around 0.27 keV, due to the C K_α_ peak, points to the absence of carbon contamination within the detection limit of the technique (around 1% atomic), even at the lower deposition temperature of 300 °C. This aspect points to the excellent thermal properties of the [VO(acac)_2_] as a V precursor and to the good optimization of the MOCVD process.

### 3.3. THz Spectroscopic Analysis

**Figure 6:**
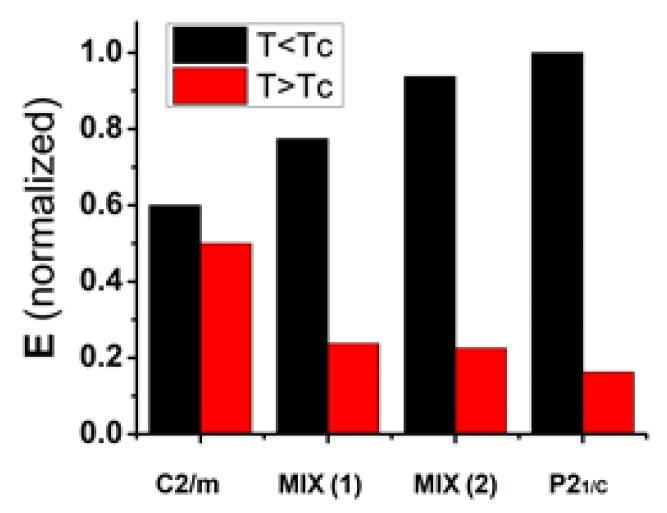
Histograms showing the phase change performances of different VO_2_ crystalline phases. In black and red, the normalized peak-to-peak amplitudes of the time-dependent THz signals getting through VO_2_ films at temperatures 22 °C ($T<T_{c}$) and 90 °C ($T>T_{c}$), respectively. The normalization has been operated employing the peak-to-peak of the P2_1_/c phase for $T<T_{c}$. MIX(1) and MIX(2) represent different mixed phases C2/m + P2_1_/c. In MIX(1), the C2/m phase dominates; in MIX(2), the P2_1_/c one prevails instead.

Preliminarily, the quality of the conducting phase of several VO_2_ films has been tested just by monitoring in time domain the change in the transmitted THz signal at the two reference temperatures: 22 °C ($T<T_{c}$) and 90 °C ($T>T_{c}$). Along with the sample showing the pure phases C2/m (VO_2_ sample on Si(100) at 350 °C, Figure 2b) and P2_1_/c (VO_2_ sample on Si(100) at 450 °C, Figure 2d), we have tested films showing mixed phases in which the C2/m and P2_1_/c components co-exist (a prototype of these films is the VO_2_ sample on Si(100) at 400 °C, Figure 2c). In Figure 6, a representative histogram of the phase change performances is reported. Black and red columns are the peak-to-peak (PtP) amplitudes for $T<T_{c}$ and $T>T_{c}$, respectively. PtP values have been normalized with respect to the highest PtP measured for the phase P2_1_/c at $T<T_{c}$. MIX(1) represents a sample with a mixed phase in which the C2/m prevails, whereas in MIX(2) the P2_1_/c phase is dominant. According to the results summarized in Figure 6, the higher the component P2_1_/c with respect to C2/m, the stronger the phase change. The pure phase P2_1_/c manifests the highest insulating/conducting transition passing through VO_2_ films below and above $T_{c}.$ Phase change performances of MIX(1) and MIX(2), representing different C2/m + P2_1_/c mixed phases, reflect the nature of the two samples. In MIX(1) the C2/m phase dominates, whereas in MIX(2) the P2_1_/c one prevails.

**Figure 7:**
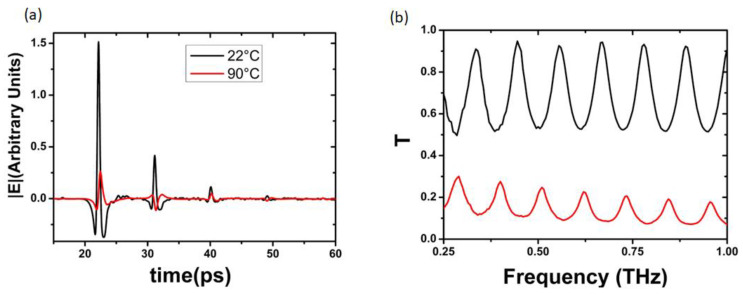
(**a**) Time-dependent THz signals and (**b**) transmissions recorded for a VO_2_ sample having the pure crystalline phase P2_1_/c below and above *T_c_* (black and red curve, respectively).

The transmitted THz signal for a film 1.2 μm thick in the pure P2_1_/c phase is reported in Figure 7b. The signal at T=22 °C (black curve) practically overlaps the signal passing through the bare Si substrate, indicating a high transparency of the sample and making difficult a reliable extraction of $\tilde{\varepsilon }.\;$ Conductivity σ values lie around zero, as shown previously [21].

At T=90 °C, the PtP amplitude of the THz signal passing through the vanadium oxide film undergoes an 80% reduction with respect to the

RT case. Moreover, the time dependence shown in the Fabry–Perot (FP) features the signature of a highly conducting state, recognizable by the upside-down behavior of the even oscillations [21].

**Figure 8:**
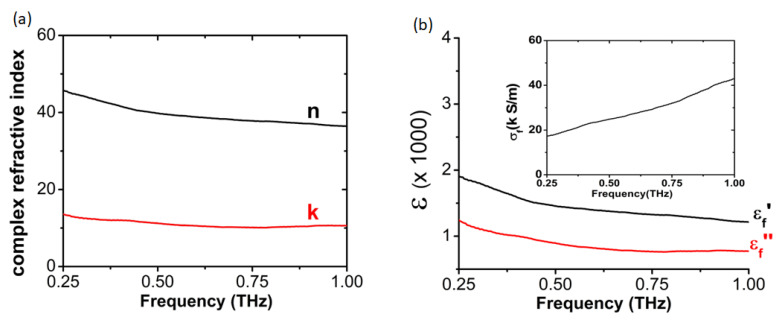
The complex refractive index (**a**) and the dielectric function (**b**) of the VO_2_ film having the pure P2_1_/c phase as a function of frequency. Inset: the film conductivity.

The complex refractive index and the dielectric function of the VO_2_ film having the pure P2_1_/c phase is reported in Figure 8. The imaginary part of ${\tilde{\varepsilon }}_{f}$ presents a decaying Drude-like behavior, but ${\varepsilon \prime }_{f}$ values are still positive, signaling that the general behavior of VO_2_ films is not fully metallic, as also recognized elsewhere [40]. The presence of a strong backscattering process [41] is also observable in the positive derivative of conductivity, as reported in the inset of Figure 8b.

## 4. Conclusions

In the present study, an optimized MOCVD process has been reported for the selective and reproducible synthesis of monoclinic VO_2_(M) P2_1_/c and/or VO_2_(B) C2/m phase in the form of thin films. The focus has been devoted to the effect of the deposition temperature in order to finely tune both the crystalline structure of the VO_2_ films and the morphology of the film surfaces. Particularly, at lower deposition temperatures, i.e., 300 °C and 350 °C, the pure C2/m phase is stabilized, and at higher temperature, i.e., 450 °C, the pure P2_1_/c has been found. The mixture of the two phases is, instead, stabilized at the deposition temperature of 400 °C. The related morphologies vary as a function of deposition temperature, from films with compact and homogenous plate-like grains to parallelepiped grains and equiaxial grains deriving from flake-like features.

Finally, the electrodynamic properties have been assessed using THz time domain spectroscopy, which allows the functional properties of the VO_2_ thin films to be shown at very high frequency. The functional validation, executed by heating the samples above the critical temperature value, i.e., *T_c_* = 78 °C, displays the conducting properties of the metallic-like phase, and their relationship with the structural features of the deposited films.

## Data Availability

Data are available from the authors upon request.
